# Formulation of food grade *Limosilactobacillus fermentum* for antifungal properties isolated from home-made curd

**DOI:** 10.1038/s41598-023-45487-4

**Published:** 2023-11-21

**Authors:** Sucheta Mandal, Narayan Chandra Mandal

**Affiliations:** 1 Mycology and Plant Pathology Laboratory, Department of Botany, Visva-Bharati, Santiniketan, 731235 West Bengal India; 2 Department of Botany, Banwarilal Bhalotia College, Paschim Bardhaman, Asansol, 713303 West Bengal India

**Keywords:** Microbiology, Molecular biology

## Abstract

Food spoilage has become a worldwide problem. *Limosilactobacillus fermentum* LAB212, isolated from home-made curd produces some potent antifungal compounds which can combat a wide range of spoilage and pathogenic fungi by disrupting their cell wall. Dual culture overlay assay and co-culture assay have confirmedly shown the potentiality of the strain. DOWEX50H ^+^ extraction and chemical characterization by high performance liquid chromatography show that lactic acid and acetic acid are playing the key roles in executing the antifungal activity. DPPH scavenging assay proves that the strain also exhibits a good antioxidant activity. After observing all the beneficial features and social need of the chemical preservative free food it is becoming highly prospective to exploite the strain commercially. In an experiment conducted for 180 days it was standardized that LAB212 supplemented with MRS and inulin is found most effective combination when challenged against the spoilage fungal species of *Aspergillus flavus* VBAH14, *Penicillium rubens* VBCA11, thus can be used as a very effective preservative agent. Using this strain as bio-preservative agent will also minimize the food borne diseases.

## Introduction

Rapidly increasing global population is constantly facing a challenge of food insecurity which is totally beyond the criteria of sustainable development. Less production of food is not only responsible for this situation but also a large portion of food, both raw and processed, is being spoiled during storage. Such waste results into a major economic loss per year^1^. According to FAO, 2014^2^ the monetary value of the waste food per year is about US $1 trillion. Developing country like India, where the economy relies upon agriculture, wastage of crops tends to lower down the overall economy of the country, so eventually there will be no improvement in poverty level. Fungal contaminations are the main reason for these spoilages. Moreover predominant fungal species like *Fusarium* sp., *Aspergillus* sp., *Penicillium* sp., *Zygosaccharomyces* spp. and *Candida* spp. during storage produce varieties of toxingenic compounds which are hazardous for human health^3^. Researchers and scientists are enormously working in this field for developing some strategies to extend the shelf life and to prevent spoilage of stored foods by treating them with heat, infrared rays, using modified atmospheres during packaging, or by adding some chemical preservatives (such as sorbic, benzoic and propionic acids)^4^. But none of these approaches came out with hundred percent successes, thus spoilage still remained a global problem^5^. On the other hand application of fungicides can control spoilage but they also exert toxic and harmful effects to human beings. To overcome this crisis a safe bio-preservation method is required. Microorganisms like lactic acid bacteria (LAB) have been used for centuries as bio-preservative agents in food industries^6^. LAB are Gram positive non-sporulating bacteria belong to the phylum *Firmicutes*, class *Bacilli* and order *Lactobacillales*^7^. They are non pathogenic having GRAS status (generally recognized as safe)^8^. LAB are mainly comprises of the genera like *Lactobacillus*, *Leuconostoc*, *Pediococcus* and *Streptococcus*, and all of them play an active role in food fermentation^9^. Beside fermentation, LAB strains show good antifungal activity which prevents the growth of spoilage microorganisms by producing lactic acid and acetic acid; moreover, they are also able to produce some other bioactive molecules, such as phenyllactic acid, fatty acids, hydrogen peroxide and bacteriocins^10^

Several studies reported about the inhibitory effect of different LAB strains on mold growth and mycotoxin production. *Lactobacillus casei* var *rhamnosus* LC-705, *L. reuteri*, *Pediococcus acidilactici*, *L. lactis*, *L. pentosus*, *L. plantarum*MiLAB14 & MiLAB 393, *L. reuteri*1100, *L*. *fermentum* C14 shows strong antifungal activity against *Aspergillus parasiticus*, *Saccharomyces cerevisiae*, *Candida lusitaniae*, *C. alicans*, *Cladosporium* spp., *A. flavus*, *Monilia* spp., *Fusarium avenaceum*, *F. gramminearum*^11–14^. Rouse et al.^15^ observed *Pediococcus pentosaceus* produced some cyclic dipeptide compound which has strong inhibitory effect against *Penicillium expansum*, *P*.*acidilactici* LAB 5 and also able to extend the shelf life of some bakery products^16^.

In the present study a potent LAB strain was isolated, characterized, and identified by 16S rDNA sequence homology. We had also checked the antifungal activity of the LAB against spoilage fungi and its application as a bio-preservative agent during storage period.

## Results

### Isolation, characterization and identification of potent LAB strain having antifungal activity

**Figure 1:**
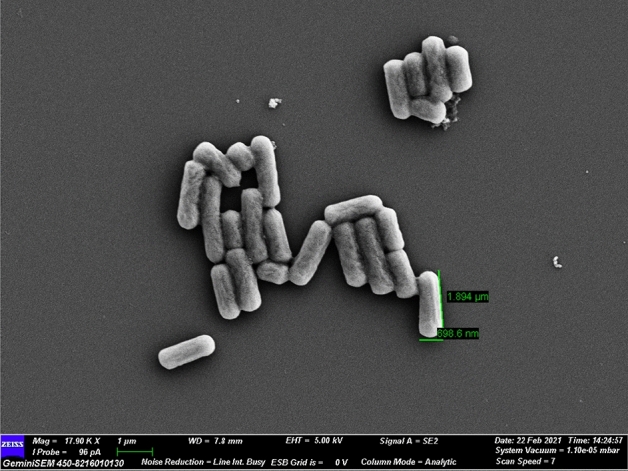
Scanning electron microscopic image of *Limosilactobacillus fermentum* LAB212.

From a number of LAB colonies four were found efficient in inhibiting fungal growth during screening. Among the four morphologically different LAB strains LAB212 exhibits the maximum antifungal activity and considered as potent one, hence selected for further studies.The strain LAB212 produces small off-white colonies on MRS plate. Light microscopic and SEM (Fig. 1) observation revealed Gram positive, non-motile, non-endospore forming rod shaped bacteria present singly. It exhibited positive result for MR & nitrate reductase, and negative against VP, catalase & indole test (Table 1). LAB212 can utilize different carbohydrates like lactose, fructose, maltose, dextrose, galactose, sucrose, salicin, inulin and ONPG as carbon source (Table 1). The LAB strain was found to be resistant to kanamycin, lincomycin, methicillin, tobramycin, vancomycin, cefaloridine antibiotics.

**Table 1:** Morphological and biochemical properties of LAB212.

Charcteristics	LAB212
Colony pigmentation	Off white
Colony shape	Flat with smooth margin
Shape of bacteria	Single rod
Gram nature	+
Endospore	Absent
Mobility	Non-motile
Methyl red	+
Voges Proskauer	−
Nitrate reductase	+
Indole	−
Catalase	−
Lactose	+
Xylose	−
Maltose	+
Fructose	+
Dextrose	+
Galactose	+
Raffinose	−
Trehalose	−
Inulin	+
Salicin	+
Sucrose	+
l-Arabinose	−
ONPG	+
Esculin hydrolysis	−

‘+’ positive reaction; ‘−’ negative reaction.

**Figure 2:**
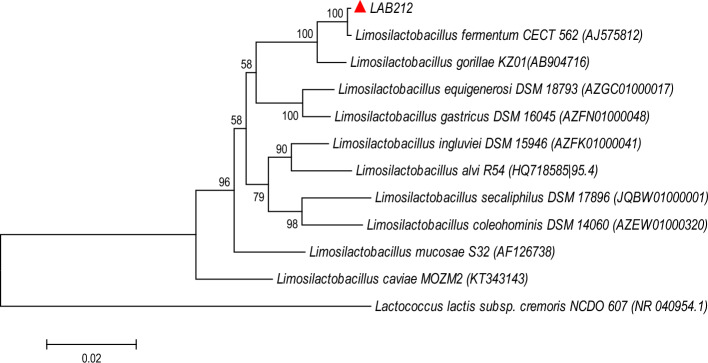
Neighbor joining phylogenetic tree based on 16S rDNA sequence of *Limosilactobacillus fermentum* LAB212 showing relationship with other members.

Maximum antagonistic activity showing strain LAB212 was identified by performing 16S rDNA sequence homology. Single discrete PCR amplicon band of ~ 1500 bp was observed on 1% agarose Gel under UV-transilluminator (Genei). After sequencing forward and reverse sequence data were aligned by multiple alignment software program ClustalO and nucleotide sequence was generated. BLAST analysis on NCBI Genebank database of this 16S rDNA sequence with Ez-Taxon database and phylogenetic analysis using MEGA5 software package following Kimura’s two parameter model the strain was identified as *Limosilactobacillus fermentum* (Accession No. MZ359838)*.* 1354 bp 16S rDNA sequence of LAB212 showed 99.92% pairwise similarities with *Limosilactobacillus fermentum* CECT 562 (AJ575812). Neighbor joining tree showed LAB212 clustered with wild type of *L. fermentum* CECT 562 (AJ575812). *Lactococcus lactis* subsp *cremoris* NCDO 607 (NR 040054.1) was used as an out group (Fig. 2)*.*

### Optimization of bacterial growth at different media and temperature

**Figure 3:**
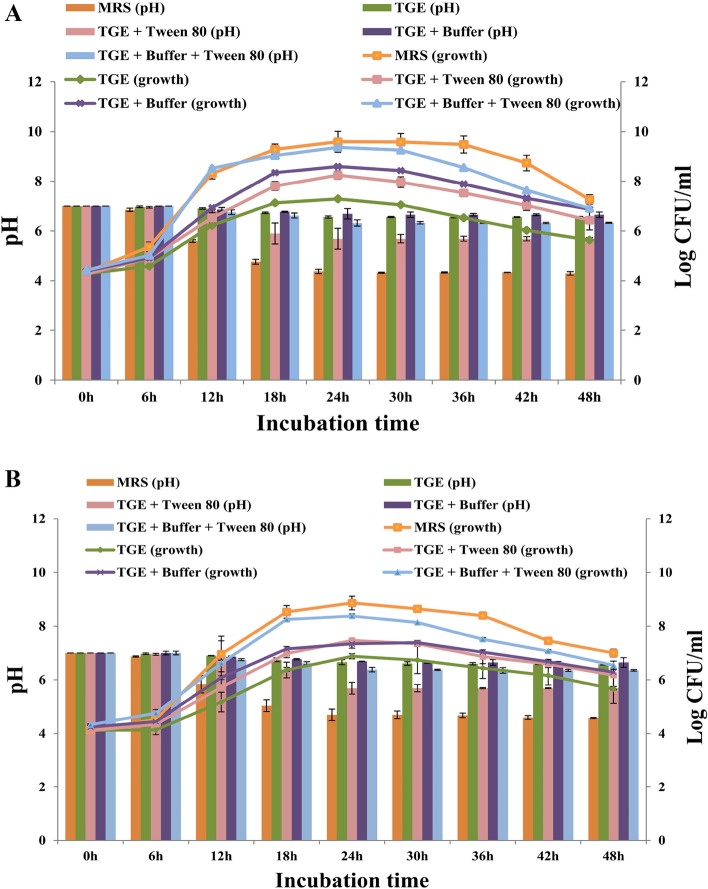
Optimization of growth kinetics along with pH reduction of LAB212 in different media viz. MRS, TGE, TGE + Buffer, TGE + Tween 80 and TGE + Buffer + Tween 80, 28 °C (**A**); 37 °C (**B**).

The growth kinetics of LAB212 was monitored in different medium at two different temperatures. All the five different mediums were inoculated with overnight grown culture of LAB212 and incubated at 28 °C and 37 °C. The lag phase continued for 8 h after incubation at 28 °C (Fig. 3A) and in 37 °C it continued upto 10 h. In both cases it was found that the bacterial cells divide more frequently in MRS and TGE + Tween80 + buffer, as a result both the growth media have small lag phase compared to the others. The bacterial growth significantly lowered down in TGE medium but addition of buffer with TGE again triggered the growth. Among all the media MRS and TGE + Tween80 + buffer shows comparable higher growth after 24 h in both temperatures, but the overall growth became hindered at 37 °C (Fig. 3B). The change in pH along with bacterial growth was also noticed. In the stationary phase the pH of MRS and TGE + Tween80 + buffer decrease upto 3.7 and 6.3 respectively.

### Antifungal spectrum of LAB212

**Figure 4:**
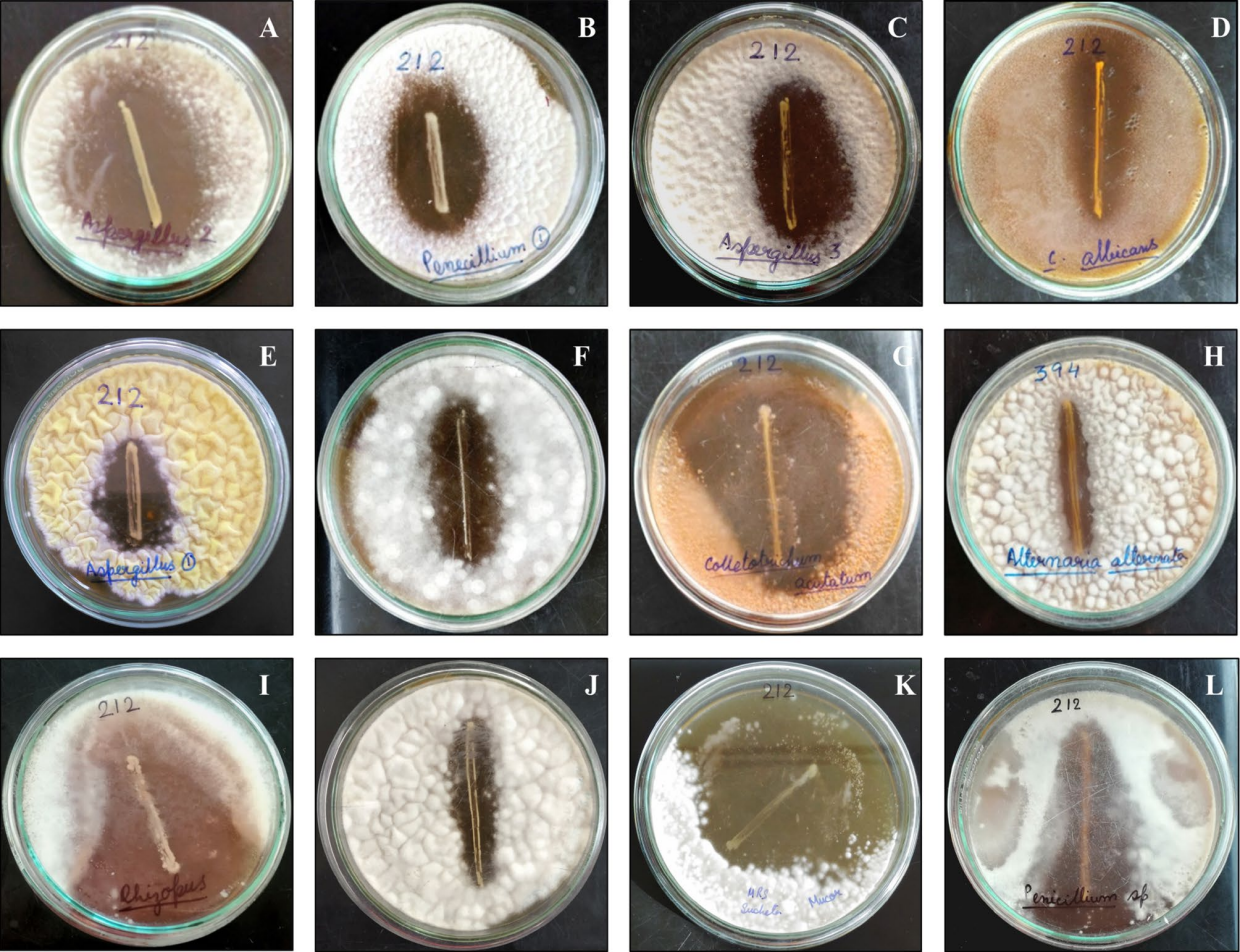
Zone of inhibition produced by LAB212 against different mycotoxigenic and human pathogenic fungi (**A**–**E**), plant pathogenic fungi (**F**–**H**) and spoilage fungi (**I**–**L**). *Aspergillus flavus* VBAH14 (**A**); *Penicillium rubens* VBCA11 (**B**); *Aspergillus flavus* MTCC2799 (**C**); *Candida albicans* MTCC183 (**D**); *Aspergillus parasiticus* MTCC2796 (**E**); *Alternaria solani* MTCC2101 (**F**) *Colletotrichum acutatum* MTCC2074 (**G**); *Alternaria solani* MTCC2101 (**H**); Rhizopus sp. VBCA12 (**I**); Aspergillus sp. VBAH10 (**J**); Mucor sp. VBBM7 (**K**); Penicillium digitatum VBCS1 (**L**).

Antimicrobial activity of *L*. *fermentum* LAB212 was checked against a number of spoilage and pathogenic microbes by dual culture overlay assay. LAB strain showed antifungal activity against five spoilage fungi and eight pathogenic fungi (Table 2, Fig. 4) but failed to inhibit plant disease causing fungi *Helminthosporium compactum* MTCC351. Clear zone of inhibition against maximum tested organisms indicates the broad spectrum antifungal activity of LAB212. Among different categories of fungi maximum inhibition was observed against *Colletrotrichum acutatum* MTCC2074 followed by *Mucor* sp. VBBM7, *A. flavus* MTCC2799, *A. flavus* VBAH14, *P. rubens* VBCA11 and *Rhizopus* sp. VBCA12. The LAB isolate can inhibit the spore formation of *Aspergillus* sp.VBAH10 even after 72 h incubation. It can effectively control the mycelial growth of aflatoxin producing strains *A. parasiticus* MTCC2796, *A. flavus* MTCC2799 and *A. flavus* VBAH14.

**Table 2:** Fungal inhibition spectrum of LAB212.

	Fungal strains	Zone of inhibition (cm.) ± SD
Spoilage fungi	*Penicillium digitatum* VBCS1	2.4 ± 0.3
	*Aspergillus* sp. VBAH10	1.5 ± 0.4
	*Mucor* sp. VBBM7	3.5 ± 0.3
	*Rhizopus* sp. VBCA12	2.4 ± 0.3
	*R. stolonifer* VBAM1	2.6 ± 0.4
Human pathogenic fungi	*P. rubens* VBCA11	3.2 ± 0.2
	*A. flavus* VBAH14	3.8 ± 0.3
	*Candida albicans* MTCC183	2.1 ± 0.1
	*A. flavus* MTCC2799	2.1 ± 0.3
	*A. parasiticus* MTCC2796	1.6 ± 0.2
Plant pathogenic fungi	*Helminthosporium compactum* MTCC351	–
	*Colletotrichum acutatum* MTCC2074	4.8 ± 0.2
	*Alternaria solani* MTCC2101	1 ± 0.2
	*A. alternate* VBAV007	1.3 ± 0.2

(−): absence of inhibition zone, (±) SD: mean of breath of zone of inhibition.

### Antifungal activity of *L*. *fermentum* LAB212 on buffered medium

**Figure 5:**
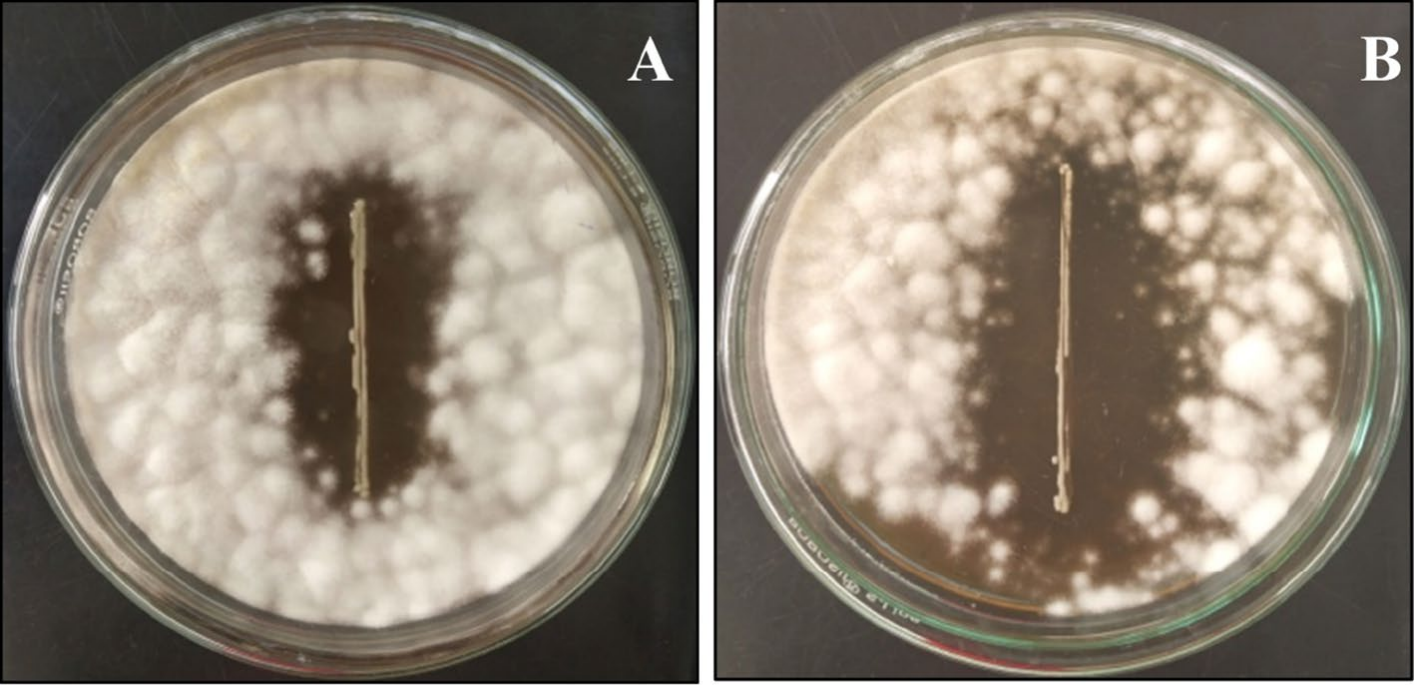
Zone of inhibition produced by LAB212 in buffer media against *A. flavus* VBAH14 (**A**); *P. rubens* VBCA11 (**B**).

Antifungal activity of LAB212 was also checked in buffered MRS medium against *A. flavus* and *P. rubens* following dual culture overlay assay. After 48 h of incubation clear zone of inhibition of 18 ± 0.2 mm and 24 ± 0.3 mm for *A. flavus* VBAH14 and *P. rubens* VBCA11 respectively were observed indicating the persisting nature of antifungal activity of LAB in buffered media (Fig. 5). Diameter of the zone has decreased slightly in buffered medium compared to the non-buffered media.

### Optimization of antifungal compound production at different media and temperature

To obtain the maximum production of antifungal compounds, the LAB isolate was grown in different medium viz. MRS, TGE, TGE + Buffer + Tween80 and overlaid with ME, Potato Dextrose, peptone, yeast extract containing fungal spores of *A*. *flavus* & *P. rubens.* Sufficient growth of the LAB isolate was observed in MRS and TGE + Buffer + Tween80 medium but growth in TGE medium was comparatively reduced. Along with the growth, the zone of inhibition varies in each case. Among all the combinations, MRS plate streaked with LAB212 and overlaid with ME soft agar showed maximum zone of inhibition at both 28 °C and 37 °C (Table 3).

**Table 3:** Antifungal spectrum of LAB212 in different media and temperature by dual culture overlay assay.

Fungal strains	Zone of inhibition produced by LAB212 ± SD							
	28 °C				37 °C			
*A*. *flavus* VBAH14	MRS + ME	MRS + PDA	MRS + YE	MRS + peptone	MRS + ME	MRS + PDA	MRS + YE	MRS + peptone
	3.6 ± 0.2	1.2 ± 0.1	–	–	2.3 ± 0.1	1.1 ± 0.3	–	–
	TGE + ME	TGE + PDA	TGE + YE	TGE + peptone	TGE + ME	TGE + PDA	TGE + YE	TGE + peptone
	–	–	–	–	–	–	–	–
	TGE + buffer + tween 80 + ME	TGE + buffer + tween 80 + PDA	TGE + buffer + tween 80 + YE	TGE + buffer + tween 80 + peptone	TGE + buffer + tween 80 + ME	TGE + buffer + tween 80 + PDA	TGE + buffer + tween 80 + YE	TGE + buffer + tween 80 + peptone
	–	–	–	–	–	–	–	–
*P. rubens* VBCA11	MRS + ME	MRS + PDA	MRS + YE	MRS + peptone	MRS + ME	MRS + PDA	MRS + YE	MRS + peptone
	3.1 ± 0.3	–	–	–	2.2 ± 0.1	1.4 ± 0.3	–	–
	TGE + ME	TGE + PDA	TGE + YE	TGE + peptone	TGE + ME	TGE + PDA	TGE + YE	TGE + peptone
	–	–	–	–	–	–	–	–
	TGE + buffer + tween 80 + ME	TGE + buffer + tween 80 + PDA	TGE + buffer + tween 80 + YE	TGE + buffer + tween 80 + peptone	TGE + buffer + tween 80 + ME	TGE + buffer + tween 80 + PDA	TGE + buffer + tween 80 + YE	TGE + buffer + tween 80 + peptone
	–	–	–	–	–	–	–	–

(−) absence of inhibition zone, (±)SD: mean of breadth of zone of inhibition.

### SEM studies of spoilage fungi

**Figure 6:**
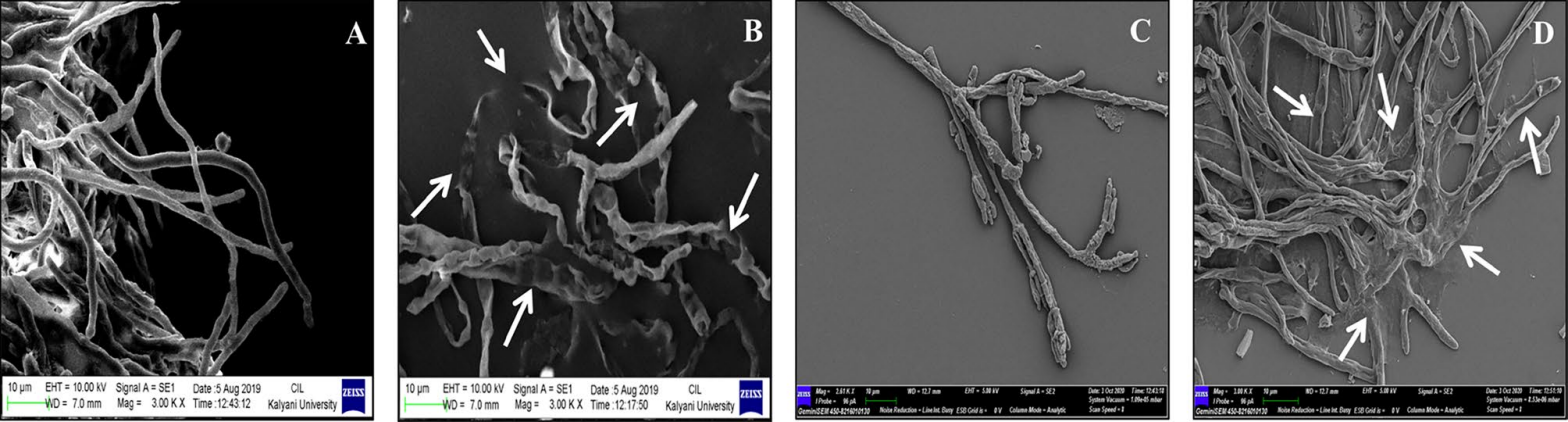
Scanning electron micrograph of toxigenic and spoilage fungi. *A. flavus* VBAH14 (**A**); *A. flavus* VBAH14 treated with LAB212 (**B**); *P. rubens* VBCA11 (**C**); *P. rubens* VBCA11 treated with LAB212 (**D**).

To check the effect of antifungal compound produced by LAB212 on fungal mycelia SEM study was performed. SEM images of the mycelia taken from zone of inhibition showed prominent degradation. Drastic distortion as well as pore formation may be the probable mechanism for fungal growth inhibition. Such mycelial disintegration is totally absent in the untreated one (Fig. 6, *A. flavus* VBAH14 (A); *A. flavus* VBAH14 treated with LAB212 (B); *P. rubens* VBCA11 (C); *P. rubens* VBCA11 treated with LAB212 (D). The result clearly indicates that, the antifungal compound produced by LAB212, acts upon the fungal cell wall and makes it porous.

### Extraction, characterization and identification of antifungal compound produced by *L*. *fermentum* LAB212

**Figure 7:**
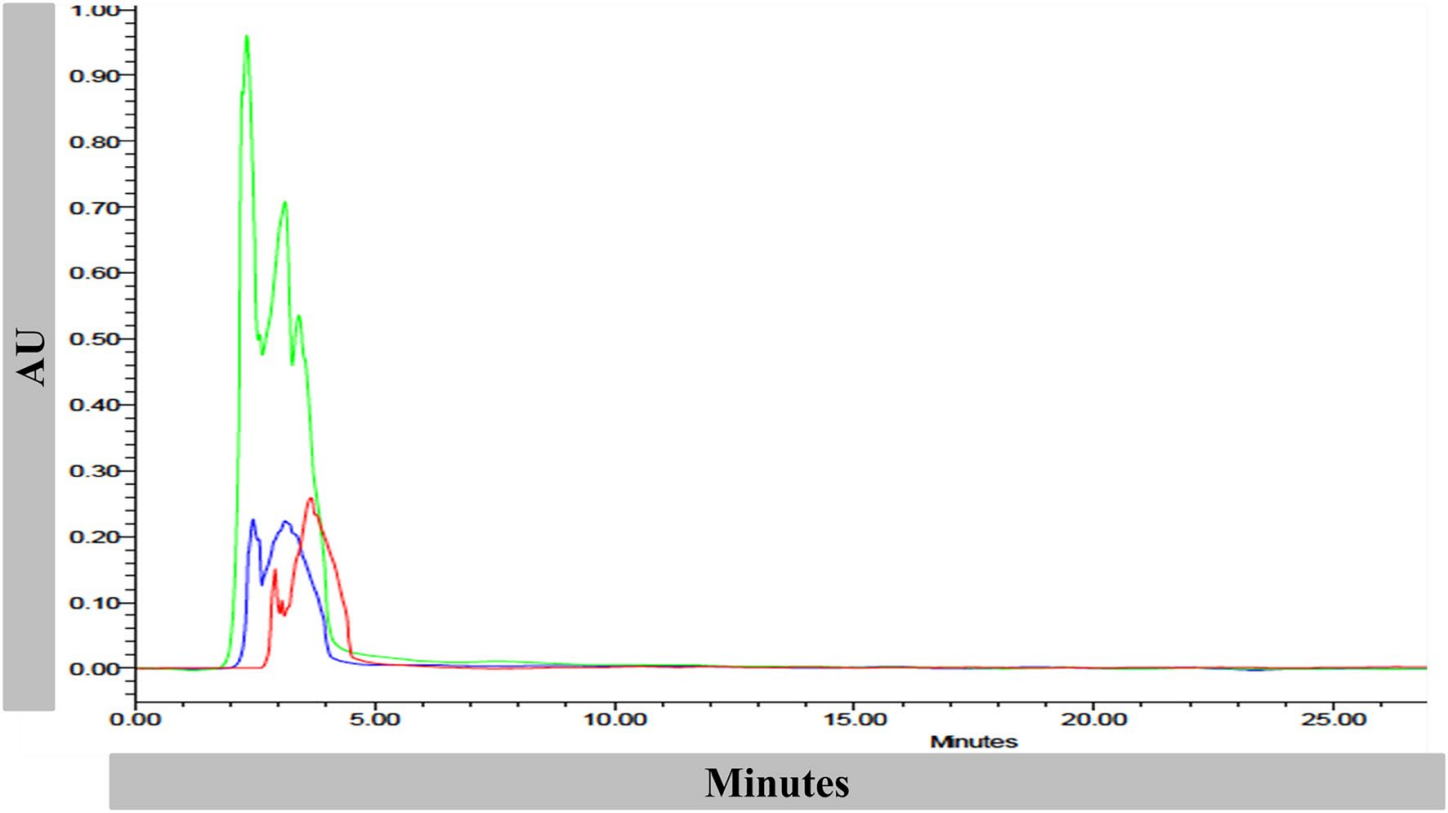
HPLC spectrum of acetic acid (red), lactic acid (blue) and DOWEX elute of *L*. *fermentum* LAB212 (green) showing retention time at 2 min and 4 min with maximum PDA absorption at 210 nm.

The bioactive antifungal compound of LAB212 was extracted by passing the CFS through DOWEX 50H^+^ (200 ± 400 mesh) column. The partially purified antifungal compound is sticky and soluble in DMSO, methanol and water. For further identification the dried DOWEX elute was subjected to HPLC along with standard lactic acid and acetic acid (Fig. 7). Appearance of sharp peak at the same retention time (RT = 2 min and 4 min) for both sample and standard suggested the presence of lactic acid and acetic acid in the crude Dowex 50H^+^ elute.

### Determination of antifungal spectrum of the compound

Antifungal activity of the DOWEX elute was checked against the spoilage fungi using agar well diffusion method. The dried elute was dissolved in DMSO at 1 mg/ml concentration and applied against *A. flavus* VBAH14 and *P. rubens* VBCA11. Prominent zone of inhibition of 13 ± 0.1 mm and 15 ± 0.2 mm for *A. flavus* VBAH14 and *P. rubens* VBCA11 respectively within 48 h incubation proves the antifungal activity of sterile lyophilisate elute. Loss of zone upon neutralization and boiling proves the acidic and heat sensitive nature of the antifungal compound.

### Study of the release of intra-cellular materials of fungal mycelia

**Figure 8:**
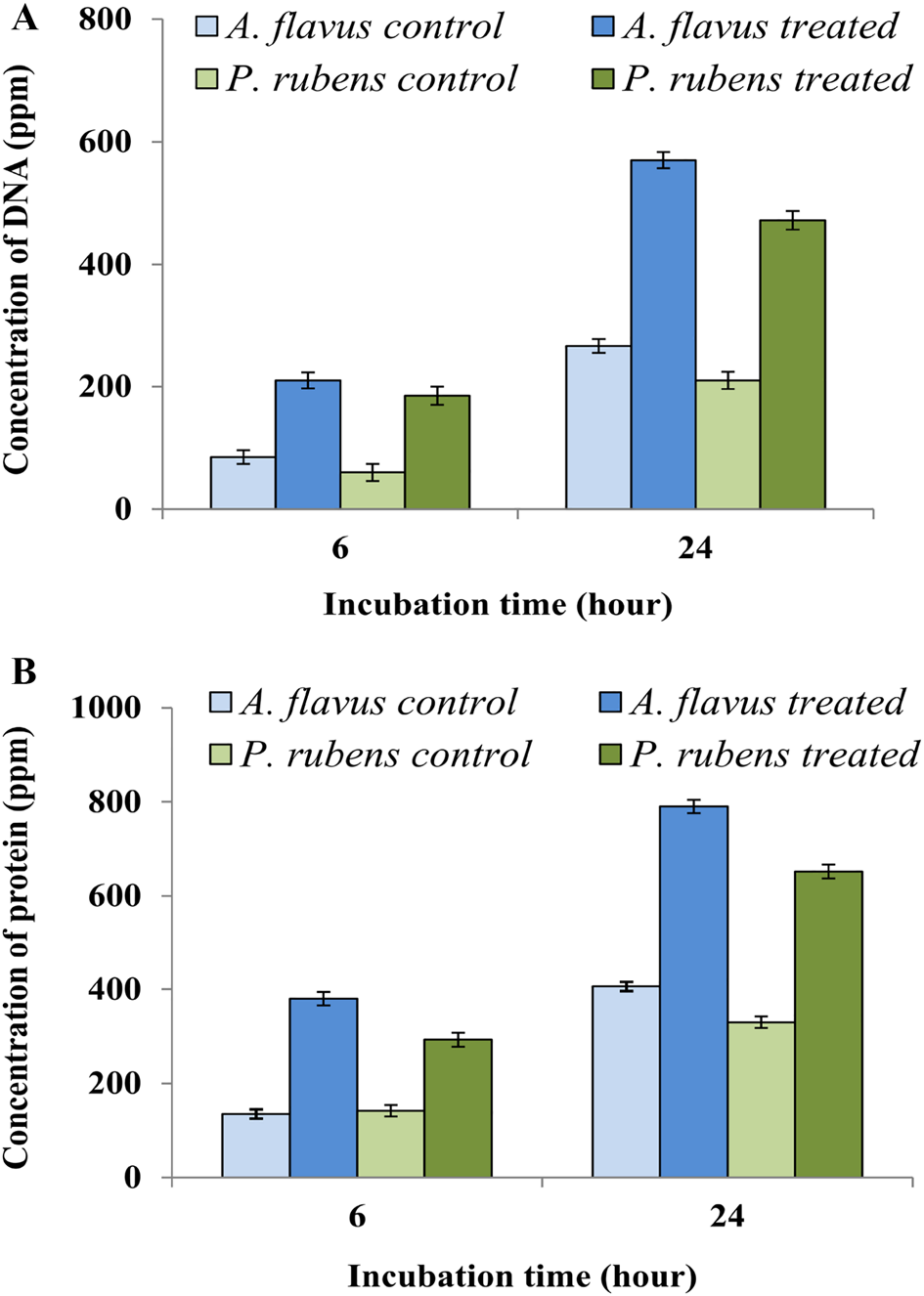
Release of intracellular materials from toxigenic fungal mycelia upon treatment with DOWEX elute of LAB212, release of DNA (**A**); release of protein (**B**).

For determining the effect of antifungal compound secreted by LAB212 on fungal mycelia extracellular concentrations of nucleic acids and protein were measured after treatment of 6 h and 24 h. Treatment with the partially purified compound showed efflux of intra-cellular proteins and nucleic acids from both the fungal mycelia. Presence of proteins and nucleic acids at the extracellular buffer within 6 h of treatment, suggested the release of intracellular material from the fungal mycelia (Fig. 8). A gradual increase in protein and nucleic acid concentration was observed after 24 h. This result also collaborates the degradation or distortion of the fungal cell wall upon treatment as observed by the previous SEM study.

### Effect of co-culture

**Figure 9:**
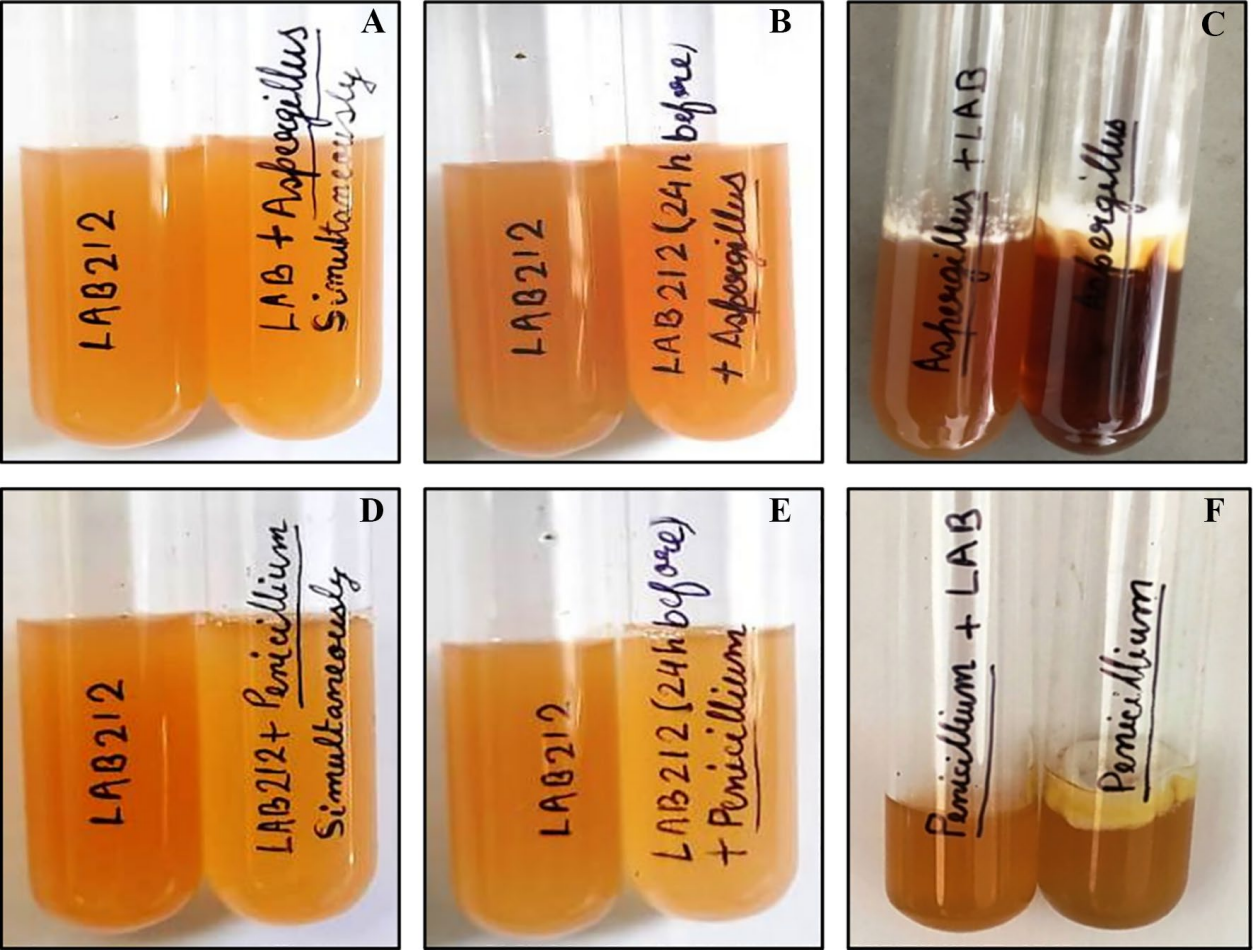
Inhibition of fungal growth by co-culture method. Growth of *A*. *flavus* VBAH14 in presence of LAB212 (**A**–**C**); Growth of *P*. *rubens* VBCA11 (**E**–**G**); LAB and fungal species grown simultaneously (**A**, **D**) (First set); LAB added 24 h before addition of fungal spores (**B**, **E**) (Second set); LAB added 24 h after addition of fungal spores (**C**, **F**) (Third set).

**Figure 10:**
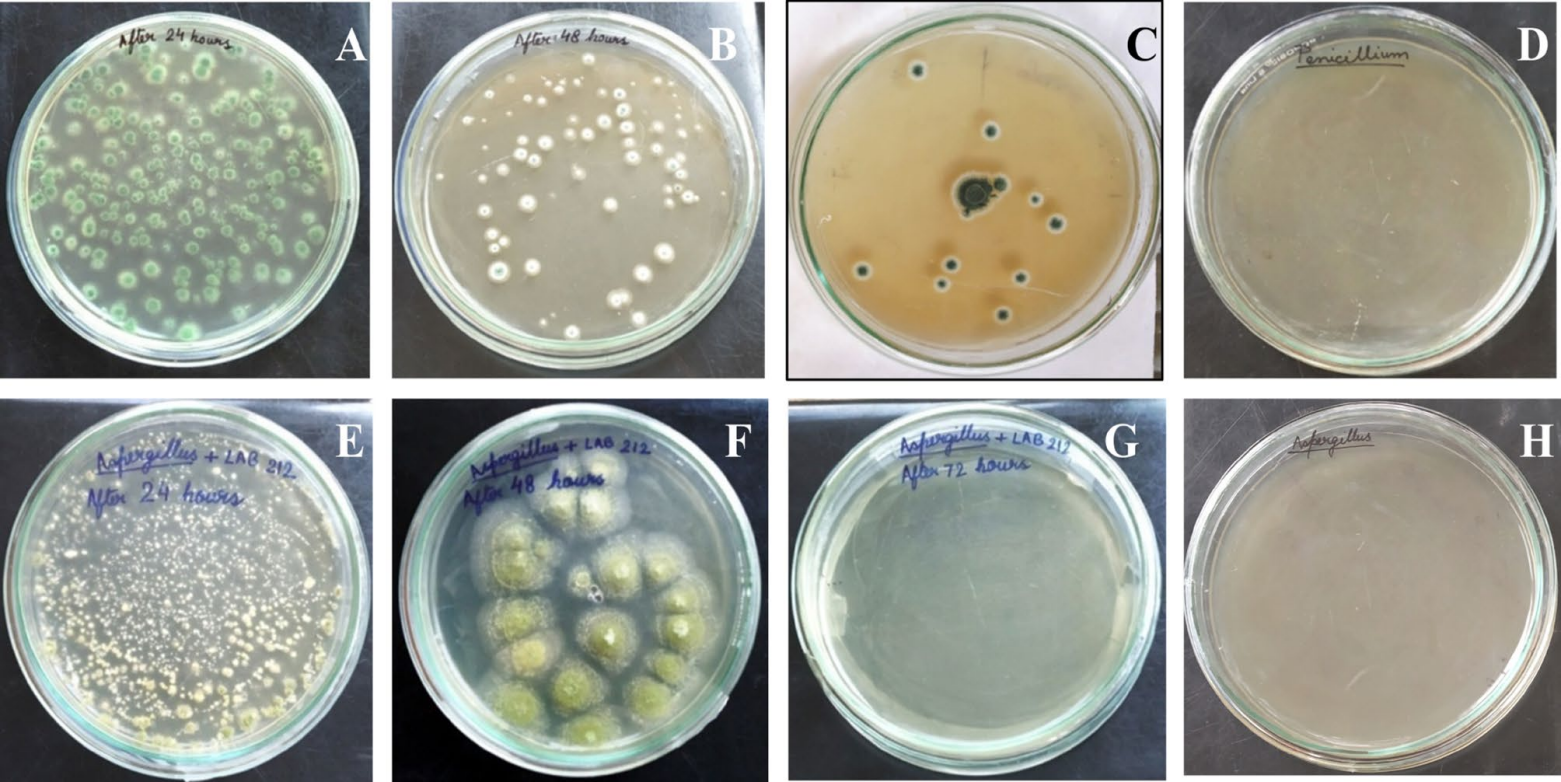
Gradual decrease of fungal cell in presence of LAB212; *P. rubens*VBCA11 (**A**–**D**) and *A*. *flavus* VBAH14 (**E**–**H**); Growth after 24 treatment (**A**, **E**); Growth after 48 treatment (**B**, **F**); Growth after 72 treatment (**C**, **G**); Growth after 96 treatment (**D**, **H**).

LAB212 and both the fungal species, influence the growth of each other when grown associatively. The growth rate of both the organisms changed in comparison to their monoculture conditions. Growth rate of LAB become slow in all the three experimental sets. In first set, no fungal growth observed where both the microorganisms were introduced together (Fig. 9A,D). The conidia of *A*. *flavus* and *P. rubens* were unable to germinate in presence of LAB but they remain alive for 72 h and 48 h respectively (Table 4), which was determined by regular spreading the broth on ME plate. After 3 days no trace of live fungal spores were found. pH of the medium was found to be 4.8 after 24 h and persists throughout the experiment. In the second set (Fig. 9B,E) where LAB was introduced earlier, it made the broth acidic (pH 4.9) and also secretes antimicrobial compounds. When the fungal conidia were introduced, they remain dormant, unable to germinate even after 48 h. On the other hand prominent fungal growth was observed in monoculture conditions after 24 h. In the last set, where the fungal spores were inoculated prior than the LAB, showed a different result (Fig. 9C,F). The spores were inoculated in MRS + ME broth, it was an ideal environmental condition for germination; hence all the spores germinated and started forming a mycelial mat like structure. But after introduction of LAB the growth rate of the fungus became lower down and tends to stop with compare to the control one. Fungal spores in this case remained alive for 3 days after LAB introduction. Growth of the microorganisms was monitored for ten days by regular spreading but no fungal spores remain alive in any sets (Fig. 10). pH of the medium was initially pH7 but a drastic drop of pH to 4.8 was observed after 24 h of addition of LAB strain.

**Table 4:** Fungal growth in presence of LAB212.

Combination of microorganisms	Time of inoculation	Presence of fungal spore			
		24 h	48 h	72 h	96 h
LAB212 + VBCA11	Simultaneous	No mycelial growth	No mycelial growth	No mycelial growth	No mycelial growth
	Fungi after 24 h of LAB inoculation	No mycelial growth	No mycelial growth	No mycelial growth	No mycelial growth
	LAB after 24 h of fungi inoculation	Mycelial growth observed	Mycelial growth reduced	Mycelial growth reduced	No mycelial growth
LAB 212 + VBAH14	Simultaneous	No mycelial growth	No mycelial growth	No mycelial growth	No mycelial growth
	Fungi after 24 h of LAB inoculation	No mycelial growth	No mycelial growth	No mycelial growth	No mycelial growth
	LAB after 24 h of fungi inoculation	Mycelial growth observed	Mycelial growth reduced	No mycelial growth	No mycelial growth

**Figure 11:**
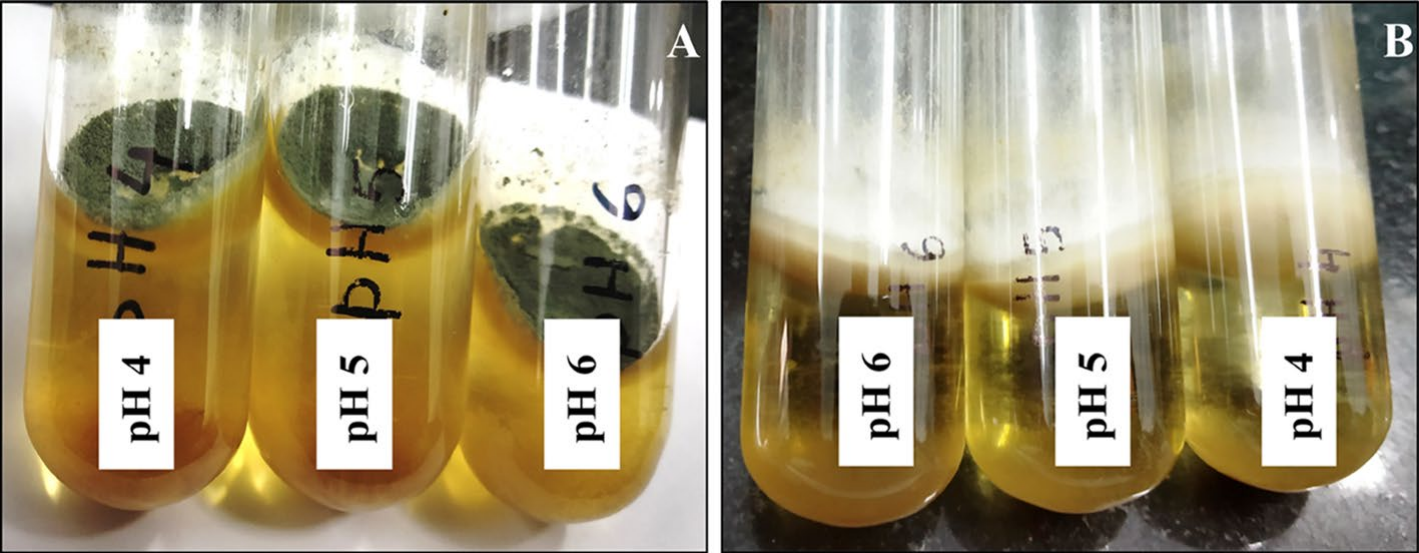
Fungal growth at different pH ranging from pH4-pH6, *P. rubens* VBCA11 (**A**); *A*. *flavus* VBAH14 (**B**).

To check the acid tolerance ability of the fungal strains, they were grown in acidic broth (pH 6, pH 5, pH 4) and observed for ten days. Prominent fungal growth was observed in all the sets (Fig. 11). Presence of mycelial growth of *A*. *flavus* was noticed in pH 6 after 48 h and for pH 5 and 4 it was visualized after 72 h. But in case of *P*. *rubens* clear mycelial growth was found after 48 h in all the sets.

### In-vitro application of LAB212 for storage of pulse and oil seeds

**Figure 12:**
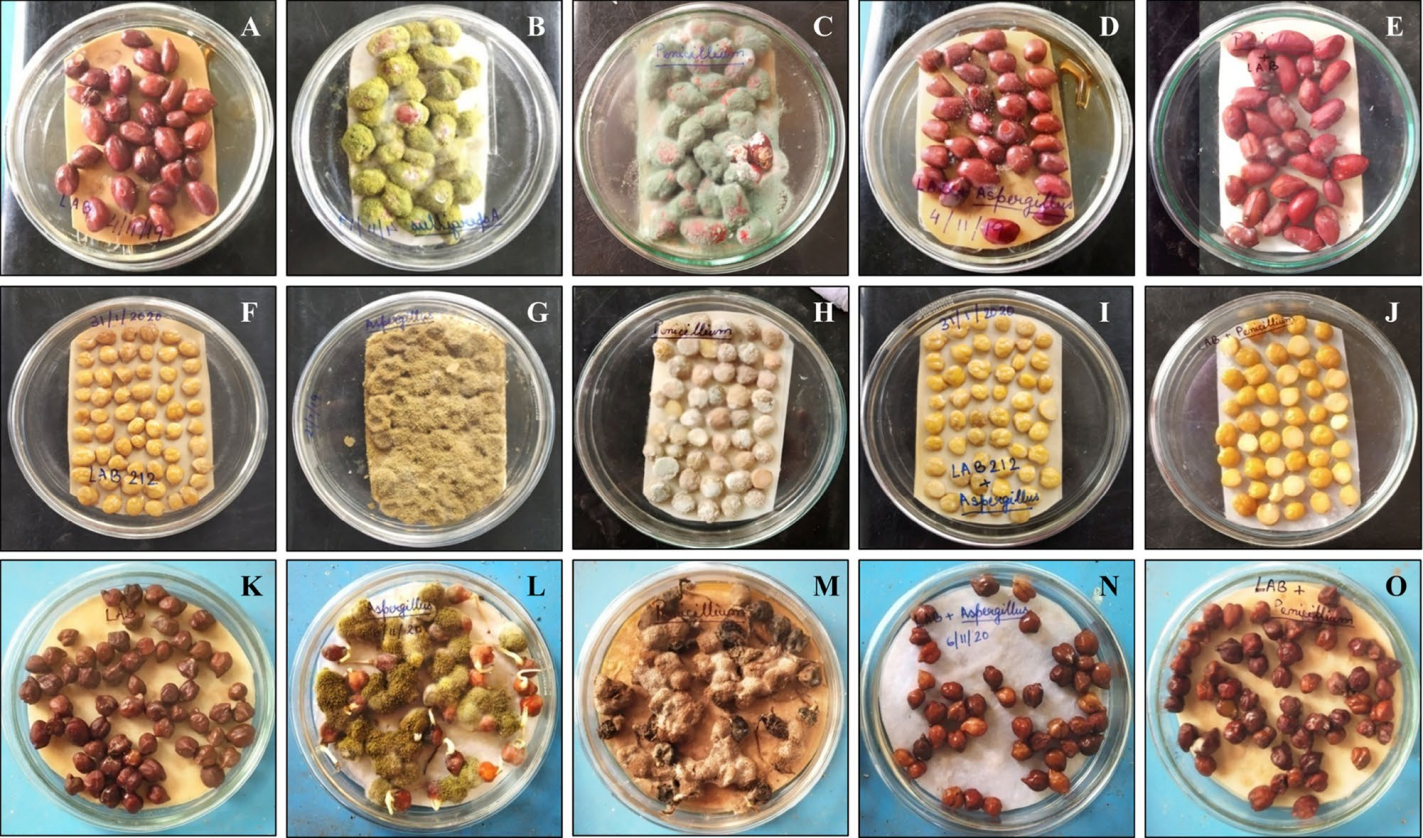
Control of spoilage of gram and ground nut seeds using *L*. *fermentum* LAB212; treated with LAB212 cell suspension (6.5 × 10^8^ CFU /ml) (**A**, **F**, **K**); treated with *A*. *flavus* (3.2 × 10^3^ spores/ml) (**B**, **G**, **L**); treated with *P. rubens* (3.5 × 10^3^ spores/ml) (**C**, **H**, **M**); treated with LAB212 cell suspension (6.5 × 10^8^ CFU/ml) and *A*. *flavus* (3.2 × 10^3^ spores/ml) (**D**, **I**, **N**), treated with LAB212 cell suspension (6.5 × 10^8^ CFU/ml) and *P. rubens* (3.5 × 10^3^ spores/ml) (E, J, O).

In the present study we wanted to observe, whether LAB strain shows its efficiency in controlling fungal spoilage in practical field or not. The experiment was done in different sets using gram and ground nut seeds for 60 days. At the end of the experiment, no spoilage was noticed in the first set (Fig. 12A,F,K), where the seeds were only treated with LAB212 cell suspension. In the second (Fig. 12B,G,L) and the third (Fig. 12C,H,M) sets vigorous fungal growth was observed from the 4th days of treatment, as a result, after twenty days the shape of the seeds became deformed, degraded and totally turned into powder. In the fourth (Fig. 12D,I,N) and the fifth (Fig. 12E,J,O) sets, where the seeds were treated with both the cell suspension of LAB212 and fungal spore, no fungal growth was observed. Seeds remained free from fungal growth and appeared fresh till the end of the experiment. Survivability of the microorganisms was checked from each set by spread plate techniques. Presence of a considerable number of LAB212 was found initially, but the CFU count starts decreasing after twenty days of treatment. After 43th day no LAB CFU was observed, showed the complete absence of LAB cells. But in case of intact gram seeds LAB CFU started to reduce from 10th day of treatment and after 24th day no LAB CFU was found. No live LAB cell was detected from any set at end of the experiment. On the other hand, viable fungal spore was observed only in second and third sets. *A*. *flavus* VBAH14 spore mixed with LAB212 cell survive in intact gram, crushed gram and ground nut seeds for 24 h, 48 h and 96 h respectively. Parallelly in case of *P. rubens*VBCA11 spores treated with LAB212 survived for 24 h, 72 h and 48 h in intact gram, crushed gram and ground nut seeds respectively.

After 60 days of treatment the unspoiled seeds were kept in ideal condition for germination in plates. Moisture level was maintained by spraying water at regular interval. But the seeds from set 1, 4, and 5 did not germinate.

### Evaluation of antioxidant activity of LAB212 by DPPH free radical scavenging assay

**Figure 13:**
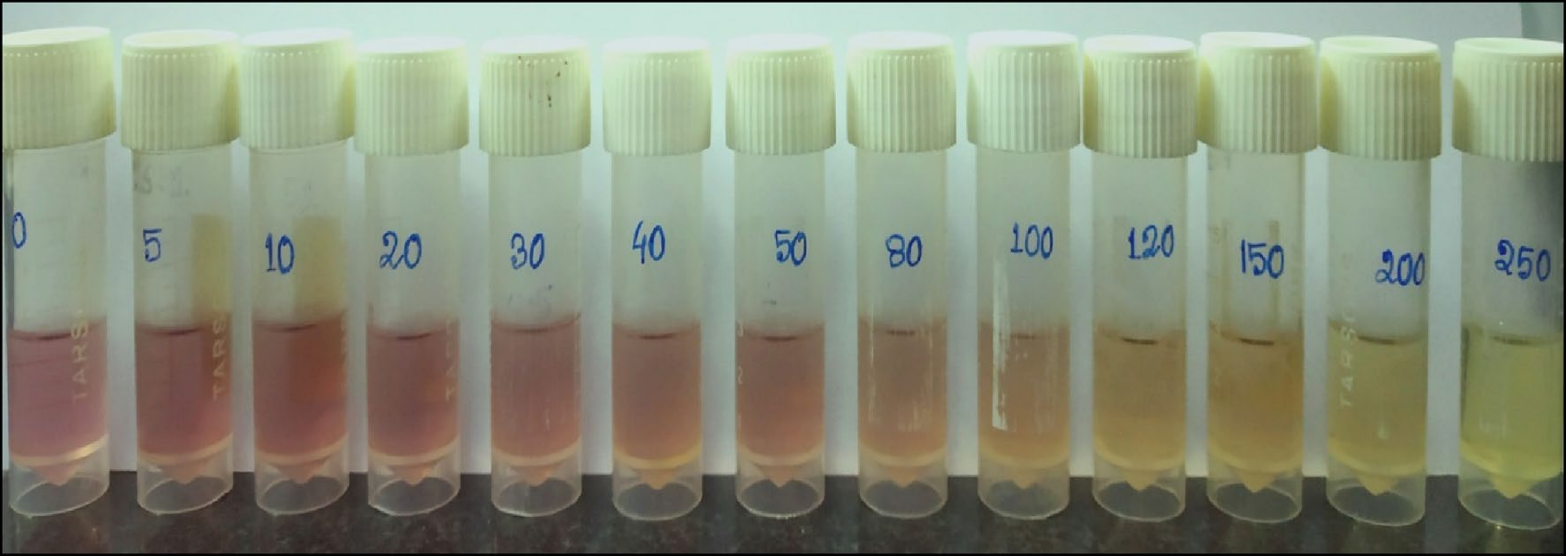
Free radical scavenging activity of concentrated methanolic extract of LAB212.

DPPH free radical scavenging assay, of the concentrated methanolic extract of LAB212 shows prominent color changes from pink to yellow (Fig. 13). Percentage of inhibiting free radical was determined by calculating IC50 value. This observation suggested the presence of antioxidant property of the concentrated methanolic extract. IC50 value of the CFS was calculated as 128 µg/ml ± 1.58 (Table 5) where as the IC50 value of ascorbic acid was 13.24 µg/ml ± 1.78, which was used as the positive control in the assay.

**Table 5:** Determination of IC50 value of LAB212 and ascorbic acid.

Concentrations (µg/ml)	Percentages (%) of inhibition of free radicals	
	LAB212 extract	Ascorbic acid (positive control)
0	0	0
5	1.204 ± 1.75	28.54 ± 1.45
10	1.927 ± 1.46	45.88 ± 1.60
20	5.783 ± 1.03	61.54 ± 2.24
30	9.638 ± 0.97	79.12 ± 2.01
40	15.667 ± 0.58	86.67 ± 3.12
50	16.867 ± 1.06	92.85 ± 2.43
80	24.096 ± 1.31	93.92 ± 1.79
100	35.903 ± 0.72	95.26 ± 3.32
120	42.650 ± 2.21	97.56 ± 2.65
150	52.530 ± 3.23	98.32 ± 2.72
200	61.445 ± 1.98	98.47 ± 1.96
250	72.566 ± 2.31	98.49 ± 2.89
IC50 (µg/ml)	128.0 ± 1.58	13.24 ± 1.78

### Standardization of the formulation using LAB212 for storage

All the useful characters of LAB212 like antimicrobial activity and anti oxidant property was already established. So to exploite the particular strain commercially, suitable formulation is required. After large scale production of LAB212 in laboratory the cells were added with sticker molecule along with some nutrition and applied for storage of gram and ground nut seeds. The total experiment was observed for 6 months (180 days). Initially the seeds were treated with LAB212 + *A*. *flavus*/*P*. *rubens* + water + CMC, LAB212 + *A*. *flavus*/*P*. *rubens* + 5 mM Na-P buffer + CMC, and LAB212 + *A. flavus*/*P*. *rubens* + MRS + CMC (Table 6). After 180 days in all the sets, seeds got spoiled due to vigorous fungal growth. LAB cells were detected for maximum 17 days. After these observations the second set was prepared where sterile silica was added instead of CMC as sticker molecule and again observed for next six months. Here seeds are added with LAB212 + *P*. *rubens* + 5 mM Na-P buffer + Silica and LAB212 + *A. flavus*/*P. rubens* + MRS + Silica and were found unspoiled till the end (Table 6). Rest of the sets was infected by fungal mycelia. In the third set, another composition was tried. Seeds were mixed with *A. flavus* / *P. rubens* along with MRS. After 180 days it was observed that all seeds remained un-spoiled. In fourth set inulin and modified MRS dissolved in Na-P buffer were added along fungal spores. In these sets also the seeds remained fresh and un-spoiled (Table 6). At the end of the experiment no LAB CFU was detected in any sets even were the seeds remain un-spoiled. But live fungal spores were found from the sets were seeds were spoiled.

**Table 6:** Different formulation of LAB212 applied on pulse and oilseeds.

Applied on	Composition	Survival of LAB212 (Day)	Survival of fungal sp.	Outcome
Gram seeds	LAB212 + *A. flavus* + water + CMC	6	180	Spoiled
	LAB212 + *P. rubens* + water + CMC	4	180	Spoiled
	LAB212 + *A. flavus* + 5 mM Na-P buffer + CMC	10	180	Spoiled
	LAB212 + *P. rubens* + 5 mM Na-P buffer + CMC	13	180	Spoiled
	LAB212 + *A. flavus* + MRS + CMC	15	180	Spoiled
	LAB212 + *P. rubens* + MRS + CMC	11	180	Spoiled
Ground nuts	LAB212 + *A. flavus* + water + CMC	3	180	Spoiled
	LAB212 + *P. rubens* + water + CMC	5	180	Spoiled
	LAB212 + *A. flavus* + 5 mM Na-P buffer + CMC	11	180	Spoiled
	LAB212 + *P. rubens* + 5 mM Na-P buffer + CMC	8	180	Spoiled
	LAB212 + *A. flavus* + MRS + CMC	17	180	Spoiled
	LAB212 + *P. rubens* + MRS + CMC	14	180	Spoiled
Gram seeds	LAB212 + *A. flavus* + water + Silica	11	180	Spoiled
	LAB212 + *P. rubens* + water + Silica	13	180	Spoiled
	LAB212 + *A. flavus* + 5 mM Na-P buffer + Silica	16	180	Spoiled
	LAB212 + *P. rubens* + 5 mM Na-P buffer + Silica	18	6	Un-spoiled
	LAB212 + *A. flavus* + MRS + Silica	21	7	Un-spoiled
	LAB212 + *P. rubens* + MRS + Silica	22	6	Un-spoiled
Ground nut	LAB212 + *A. flavus* + water + Silica	11	180	Spoiled
	LAB212 + *P. rubens* + water + Silica	13	180	Spoiled
	LAB212 + *A. flavus* + 5 mM Na-P buffer + Silica	14	180	Spoiled
	LAB212 + *P. rubens* + 5 mM Na-P buffer + Silica	19	8	Un-spoiled
	LAB212 + *A. flavus* + MRS + Silica	23	4	Un-spoiled
	LAB212 + *P. rubens* + MRS + Silica	21	7	Un-spoiled
Gram seeds	LAB212 + *A. flavus* + MRS	33	6	Un-spoiled
	LAB212 + *P. rubens* + MRS	39	4	Un-spoiled
Ground nut	LAB212 + *A. flavus* + MRS	45	7	Un-spoiled
	LAB212 + *P. rubens* + MRS	37	5	Un-spoiled
Gram seeds	LAB212 + *A. flavus* + MRS(modified) (dissolved in 5 mM Na-P buffer) + inulin	64	7	Un-spoiled
	LAB212 + *P. rubens* + MRS(modified) (dissolved in 5 mM Na-P buffer) + inulin	66	5	Un-spoiled
Ground nut	LAB212 + *A. flavus* + MRS(modified) (dissolved in 5 mM Na-P buffer) + inulin	67	6	Un-spoiled
	LAB212 + *P. rubens* + MRS(modified) (dissolved in 5 mM Na-P buffer) + inulin	69	6	Un-spoiled

## Discussion

Several LAB strains were isolated from home made curd, but, only four of them showed antifungal activity against *Mucor* sp. VBBM7. *Mucor* sp. was selected for initial screening as it is a common spoilage fungi and there are several reports of complete inhibition of this fungi by different LAB strains. Among these four strains LAB212 was selected as the most potent one for further studies, based on its broad antifungal spectrum. The strain was identified as *Limosilactobacillus fermentum*, using 16S rDNA sequence homology. Researchers had already reported about the antimicrobial activity of various LAB strains viz*. L. brevis*, *L. casei*, *L. plantarum* and *L. rhamnosus*^29,30^.

Optimization of growth temperature experiment shows that for LAB212 favorable growth temperature is 28 °C in MRS and TGE + Tween80 + buffer medium. Previous report also suggested the best growth of different LAB species like *Lactococcus lactis subsp. hordniae*, *L. lactis subsp. lactis H-559* at 28 °C along with maximum bacteriocins production^14^. Presence of growth in low pH at 28 °C and at 37 °C indicates it as both acidophilic and mesophilic bacterium. Decrease in pH along with the increasing CFU count in log phase suggested the direct correlation between cell growth and acid secretion. TGE medium alone was not suitable for bacterial growth but addition of buffer and tween80 had overcome this barrier. Tween80 increased the cell viability by reducing the metabolic pressure-induced loss and also down regulates the fatty acid biosynthesis by increasing the level of oleic acid and cyclo-propane fatty acid in the cell membrane^31^. Supplementation of Tween80 with mMRS medium enhances the growth of *L. plantarum* TMW 1.708 and *L. delbrueckii*^31^. Presence of buffer in the medium maintains the broth pH, and effectively influences the bacterial growth. LAB212 exhibits strong antifungal effect against a certain number of spoilage and pathogenic bacteria. Azole drug resistant *Candida* spp., mainly responsible for hospital-acquired contagions, can be inhibited by applying *L. plantarum* and *L. curvatus*^32^. *L. plantarum*, *L. pentosus*, *L. paracasei* shows strong antifungal effect against *Colletotrichum gloeosporioides*^33^.

Decrease in zone of inhibition in buffer media compared to the non buffered medium during overlay assay suggest that organic acids was not solely responsible for antifungal activity, some non acidic compounds were also present in the antifungal compound mix produced by LAB strain. It was assumed that the compound might be a mixture of acidic and non acidic compound. As buffered media withstand the decrease of pH a decrease in zone of inhibition was observed.

Presence of clear inhibition zone, in combination of MRS *vs* ME and MRS *vs* PDA suggested the secretion of antifungal compound. Absence of zone in other overlay constituent indicates no influence in production of antifungal compound.

SEM images clearly showed a massive disintegration of fungal cell wall by formation of pores on hyphal surface supports strong cidal effect of the antifungal compound. Similar kind of fungal cell wall distortion was observed by Ghosh et al.^22^ during studying the antagonistic activity of *Lactococcous lactis* subsp. *lactis* LABW4 on jackfruit spoilage fungi *R*. *stolonifer* VBAM1. Massive disintegration of bread mould *Mucor* sp. VBBM7 and human pathogenic *Trichophyton rubrum*MTCC297 upon treatment with *Lactobacillus fermentum* C14 was also reported by Barman et al.^14^.

HPLC of the antifungal compound secreted by LAB showed the presence of lactic acid and acetic acid. Production of lactic acid and phenyl lactic acid by *L*. *lactis* subsp. *lactis* LABW4 as main antifungal compound was reported by Barman et at.^14^. Heat stable acidic metabolite produced by *L*. *casei* CRL 239, *L*. *plantarum* (CRL 142, CRL 681, CRL 142), *L*. *fermentum* (CRL 220, CRL 236, CRL 251), *L*. *reuteri* CRL 1098, *L*. *acidophilus* CRL 1070 was effective against series of fungal species like *A*. *niger* CH101, *Penicillium* sp. CH102, *F*. *graminearum* CH103, *Geotrichumcitri-aurantii* INTA1 and *P. digitatum* INTA2. HPLC analysis of the antifungal compound showed the presence of lactic, acetic, and phenyllactic acids as active compound^34^. Mixture’ of caproic acid, propionic acid, butyric acid and valeric acid in the CFS of *L*. *sanfrancisco* CBI was also documented^35^. Apart from organic acids some proteinaceous compounds secreted by different LAB species also posses strong antifungal activity.

Application of the active compound against the fungi shows the presence of protein and DNA in culture broth, indicating the leakage and breakage on fungal mycelia.SEM images also indicate the same. Addition of the metabolite of endophytic fungus *Alternaria alternata* AE1 degrades the cell wall of *E*. *coli* and *L*. *monocytogenes*^36^.

Finally the growth pattern of fungi in presence of LAB212 was checked by co-culture method. At the end of the experiment complete inhibition of fungal strain in presence of LAB was observed in all the sets. In the first and second experiment set as the LAB strain was added simultaneously or before addition of fungal spore antifungal compound secretion starts at log phase thereby completely inhibit the germination of fungal spores. But in the set three the growth of fungal mycelia was stopped after initial mycelial mat formation. From such observations it can be said that presence of LAB can inhibit fungal growth at any stage, even after formation of fungal mat. Coallier-ascah and Idziak^37^ also observed a similar result during studying the interaction between *Streptococcus lactis* and *Aspergillus flavus*. pH of all the sets showed acidic nature of the broth, but it cannot be claimed that fungal inhibition is mainly due to presence of acids. In these sets maximum pH drops upto 4.9 but prominent growth of both the fungal species was noticed even at pH 4. Presence of inhibition zone in buffered media indicates the same. Wiseman and Marth^27^ while studying the effect of *S*. *lactis* on *A*. *paraciticus* suggested that in presence of high concentration of lactic acid growth of the fungal member was inhibited but our study does not showed complete similarity with them. Penalva and Arst, ^38^ stated that fungi can survive in acidic condition by activating the signaling cascades Pal/Rim alkaline response pathway. Phosphate solubilizing strain *A. tubingensis* and *A*. *carbonarius* can grow upto pH 2.3^39,40^.

Presence of superoxide anions, free radicals are responsible for oxidative damage causing cancer, cirrhosis, arthritis, emphysema, atherosclerosis, ischemia etc. in living organisms^41^. Catalase and hydroperoxidase enzymes convert the radical to nonradical form in human system, but presence of excessive free radial suppresses the immune system antioxidants by changing the gene expression. So nowadays people consume some synthetic antioxidants like butylated hydroxy anisole (BHA), butylatedhydroxy toluene (BHT), tertiary butylatedhydroquinon and gallic acid esters, but all had negative health effect^42^. This food grade LAB strain showed a good antioxidant activity along with its antifungal activity.

Observations of the bio-preservative potentiality of LAB212 indicate its possible use to prevent spoilage by various spoilage causing fungal and bacterial microorganisms. It was found to have the ability to inhibit the growth of *A*. *flavus* VBAH14 and *P. rubens* VBCA11 from the surface of gram and groundnut seeds. *L*. *fermentum*C14 was reported for controlling the growth of *Mucor* sp. and *B*. *subtilis* on bread surface^14^. *L. pentosus* G004, *L. fermentum* Te007, *L. paracasi* D5 can control the bread spoilage mould *A. oryzae*, and tomato puree spoilage mould *A. niger.* Surviving on butter, cheese, or on bread surface is quiet easier for any LAB strain as they can easily get their nutrition from these dairy or bakery products, but existing on pulse or oilseed surface is quiet challenging. Application of different formulation on gram and groundnut seeds shows presence of considerable number of LAB212 till 69th day.

*Limosilactobacillus fermentum*LAB212, isolated from home made curd, showed very good antifungal activity against both spoilage, pathogenic and toxingenic fungi. The partially purified antifungal compound is heat sensitive and acidic in nature. This potent bacterial strain shows good antioxidant activity and was also capable to inhibit the spoilage of gram and ground nut seeds. Suitable formulation and application of this bacterium, as a biopreservative agent will be reducing successful in at that some percentage of spoilage and thus this food grade organism can be considered as a prospective agent in the biopreservation of food particularly various kinds of edible seeds.

## Materials and methods

### Microorganisms and media

All the LAB isolates were cultured in *Lactobacillus* MRS medium (HiMedia, Mumbai, India)^17^ and stored for long term in 10% glycerol skimmed milk at 4 °C. Cultures are stored in MRS slant for regular use. Spoilage and pathogenic strains used in antifungal assay were procured from Microbial Type Culture Collection (MTCC), IMTECH, Chandigarh, India and were maintained in Malt extract (ME) agar medium (HiMedia, Mumbai, India) in slants at 4 °C as per MTCC guidelines. Other fungal strains, like, *Mucor* sp. VBBM7, *Aspergillus flavus* VBAH14, *Penicillium rubens* VBCA11, *Rhizopus stolonifer* VBAM1 and *P*. *digitatum* VBCS1 were isolated in our laboratory from spoiled bread samples, spoiled groundnut, spoiled gram seeds, rotten surfaces of jackfruit and spoiled oranges respectively. The fungal strains were identified by performing 28S rDNA sequence homology.

### Sampling and isolation

Homemade curd samples using different milk sources were collected from Durgapur, West Bengal. All the samples were immediately stored in refrigerator (4 °C) before further studies. For isolation of LAB strains, the curd samples were serially diluted and spread on *Lactobacillus* MRS plate and incubated for 48 h at 28 °C. To avoid fungal contaminations MRS plates were amended with antifungal antibiotic griseofulvin (100 µg/ml). A total four LAB strains viz. LAB211, LAB212, LAB214, LAB216 were initially selected based on their colony morphology and were also screened for antifungal activities against *Mucor* VBBM7 by dual culture overlay method^18^. Colonies showing prominent zones of inhibition against most of the tested pathogens were selected for further studies. For pure culture, LAB strains were repeatedly streaked on *Lactobacillus* MRS plate and stored for more studies.

### Characterization and identification of LAB212

LAB212 was characterized both microscopically and biochemically. Morphological characterization was done by light microscope and scanning electron microscope (SEM). Antibiotic sensitivity profile was performed by using HiMedia antibiotic octadiscs, Carbohydrate utilization property of the strain was studied using HiMedia HiCarbohydrateTM Kit (KB009) and other biochemical tests were performed following manufacturer’s guidelines of HiMedia (Mumbai, India).

Molecular identification was done by using 16S rDNA sequence homologies and phylogenetic analysis.16S rDNA was amplified with the primer (16SF and 16SR) using Veriti® 96 wellThermal Cycler (Model No. 9902). A single discrete PCR amplicon band of 1500 bp was observed on 1% Agarose Gel. Concentration of the amplicon was checked in a Nanodrop ND 8000. Purified amplified product was subjected to Bi-directional DNA sequencing reaction with forward and reverse primers using SureExtract PCR cleanup/Gel extraction kit (Genetix) in ABI 3730xl cycle sequencer. Forward and reverse sequences were assembled and contig was generated after trimming the low quality bases. The sequence analysis was carried out using Bioinformatic tool BLASTn of NCBI. Based on maximum identity score, first few sequences were selected and aligned using multiple sequence alignment software ClustalO. The evolutionary relationship was established using the Neighbor-Joining method^19^ and Phylogenetic tree was constructed by using MEGA5^20^. The evolutionary distances were computed using the Kimura 2-parameter model^21^. *Lactococcous lactis subsp. cremoris* NCDO 007(NR 040054.1) used as an out group member during phylogenetic analysis.

### Optimization of growth in different media at different temperatures

To optimize the growth condition, LAB212 was cultured in different media viz. MRS, tryptone glucose yeast extract (TGE), TGE + Tween 80 (0.5%), TGE + buffer (containing 0.2% sodium acetate, sodium citrate and dipotassium hydrogen phosphate) and TGE + Tween 80 + buffer and are separately incubated at 28 °C and 37 °C. Bacterial growth and pH change in medium were monitored in each set at every three hours interval for 48 h. Bacterial growth was checked by both, measuring the O.D. at 620 nm and counting the colony forming units (CFU). The change in medium pH was measured by digital pH meter (Systronics 802).

### Evaluation of antifungal activity

The antifungal activity of the LAB212 was checked against spoilage as well as pathogenic fungi viz. *Aspergillus flavus* VBAH14; *Penicillium rubens* VBCA11; *Aspergillus flavus* MTCC2799; *Candida albicans* MTCC183; *Aspergillus parasiticus* MTCC2796; *Alternaria solani* MTCC2101; *Colletotrichum acutatum* MTCC2074; *Alternaria solani* MTCC2101; *Rhizopus* sp. following dual culture overlay method^18^. MRS plates were first streaked with overnight grown LAB culture and incubated for 24 h at 28 °C. Plates were then overlaid with 7 ml malt extract soft agar (2% ME and 0.7% Agar) containing 10^3^ fungal spores per ml and reincubated for next 2–3 days. Antifungal activity of the LAB strain was observed, and the inhibition zones were measured.

### Antifungal activity of LAB on buffered medium

The antifungal activity of LAB isolate was checked in buffered media following dual culture overlay assay^18^ against the spoilage fungi. Addition of sodium phosphate buffer with MRS media can withstand the decrease of pH of medium. Appearance of any zone of inhibition was monitored after incubation for 48 h at 28 °C.

### Optimization of antifungal activity in different media at different temperatures

To obtain the maximum production of antifungal compound LAB212 was streaked in different media viz. MRS, TGE, TGE + Buffer + Tween80 and incubated at 28 °C and 37 °C for 24 h. All of these plates were overlaid with ME, potato dextrose, peptone and yeast extract, containing fungal spores. After 2–3 days incubation zone of inhibition was measured in each combination.

### Scanning electron microscopic (SEM) studies

SEM study along with all the further studies were performed on *A*. *flavus* VBAH14 and *P*.*rubens* VBCA11 because these two strains shows spoilage activity as well as in another study it was observed that both of them were potent aflatoxin producer. For evaluation of antifungal effect of LAB212 treated and untreated mycelia of both the fungal strains were selected. Mycelia taken from zone of inhibition interface were considered as treated and from uninhibited portion were used as controlled one. Both the mycelia were prefixed using 2% glutaraldehyde (Merck Germany) in 50 mM Na–P buffer (pH 6.5) plus 5% dimethyl sulphoxide (DMSO) (Merck, Germany) for 30 min and post fixation was done using osmium tetraoxide after washing with sterile water. Then it was passed through the series of ethylalcohol grades starting from 30 to 100%, retaining them for 10 min in every dilution for complete dehydration. Now the complete dehydrated mycelia were gold coated using an ion sputter (Coater IB-2, Gikeengeering, Japan) and observed under scanning electron microscope (Zeiss) following the standard method^22^.

### Extraction and characterization of antifungal compound

LAB212 was cultured in MRS broth at favorable growth condition. Cell free supernatant (CFS) were prepared by harvesting the cells at 10,000 rpm for 10 min and it was filtered through 0.22 μm syringe filter (Merck, Germany). Then the sterile CFS was passed through DOWEX50H^+^ (200 ± 400 mesh) column^23^ and finally lyophilized.

pH of the crude elute was checked to determine the nature of the compound. To find out the thermostability of the compound, the elute was kept in boiling water bath for 10 min. On the other hand pH of the crude elute was adjusted to7 and applied against the spoilage fungi. Uninoculated MRS broth was used as negative control.

### Determination of antifungal spectrum of the compounds

Antifungal activity of the compound was determined by performing agar well diffusion assay. The dried lyophilized DOWEX elute was dissolved in sterilized undiluted DMSO and applied against *A*. *flavus* VBAH14 and *P*.*rubens* VBCA11 after passing it through 0.22 μm PVDF membrane filter (Millex® GV, Millipore). Zone of inhibition was observed after 48 h of incubation at 28 °C.

### Identification of the antifungal compound

Chemical composition of the antifungal compound was determined by performing HPLC. The dried DOWEX elute was diluted by adding HPLC grade water and subjected to HPLC (Waters 1525 Binary system) equipped with Waters 2998 photodiode array (PDA) detector at 210 nm. 20 μl of antifungal compound along with standard acetic acid and lactic acid (Merck, India) were filtered (pore size 0.2 micron) and injected into HPLC system through Rheodyne injection valve and separation was carried out by Symmetry C18 (5 μm 4.6 × 250 mm) column with Symmetry guard column using 0.01 mol/l sulfuric acid as mobile phase, with a flow rate 0.5 ml minute^−1^^24^. Elute was collected manually through Waters Fraction Collector III and lyophilized.

### Study of the release of fungal intracellular protein and nucleic acid

In order to determine the effects of the antifungal compound of LAB212 on fungal mycelial integrity, extracellular concentrations of nucleic acids^25^ and protein^26^ was measured. *A*. *flavus* & *P. rubens* were grown in 200 ml ME broth in 500 ml Erlenmeyer flask for 48 h and then the mycelia were filtered from the broth. Now the mycelia were washed with 50 mM sodium phosphate buffer and resuspended in the same buffer. Mycelia of the both fungi were treated with the antifungal extract of LAB212 (500 μg/ml). Release of protein and DNA was quantified from the supernatants after harvesting the mycelia by centrifugation (10,000 rpm) at 6 h and 24 h.

### Inhibition of fungal growth by co-culture method

To observe the growth pattern during co culture, *A*. *flavus* VBAH14 (1.8 × 10^3^ spores/ml), *P*. *rubens* VBCA11 (1.5 × 10^3^ spores/ml) and LAB212 (2 × 10^9^ CFU/ml) strains were inoculated in ME + MRS (1:1 v/v) broth^27^. This experiment was performed in three different conditions. In the 1st set, spore suspension of *A. flavus* VBAH14 was mixed with LAB212 and introduced to culture broth simultaneously. In the 2nd set LAB cells were added in culture media 24 h before addition of VBAH14 spore and in the 3rd set spores of VBAH14 was introduced 24 h prior of addition of LAB212. Similar sets were prepared for *P*. *rubens* VBCA11 and LAB212. Monoculture condition of LAB212 and fungal sp. were used as control sets. All the experiment sets were incubated at 28 °C for ten days. To justify the result, acid tolerance capability of both the fungal strains were also checked by growing them at various pH ranging from 4 to 6. The population of LAB was measured spectrophotometrically (Shimadzu, UV_-1700_^Pharmaspec^), using uninoculated ME + MRS as blank. The population of fungal spores was measured by spore count method. Growth pattern of both the organisms were monitored till ten days.

### In-vitro control of spoilage of pulse and oil seeds using LAB212

The antifungal activity of LAB212 was applied to control the spoilage of gram (B-115) and ground nut (TAG-24) seeds in in-vitro system against the spoilage caused by *A*. *flavus* and *P. rubens.* LAB212 was grown in 200 ml MRS broth for 24 h and cells were harvested by centrifugation at 5000 rpm for 10 min at 4 °C. The CFS was discarded and the cell pellet was resuspended in 5 mM sodium phosphate buffer at concentration of 6.5 × 10^8^ CFU/ml. On the other hand spore suspension of both the fungi was prepared by suspending the spores (3.2 × 10^3^ spores/ml) in sterilized distilled water. 20gm of ground nut seeds were surface sterilized by using 4% sodium hypochlorite and placed in five sterilized petriplates. The first set was treated only with the cell suspension of LAB212; second and third sets were separately inoculated with *A*. *flavus* and *P. rubens* spore suspension. In fourth and fifth sets the cell suspension of LAB212 was mixed with both the fungal spore suspension separately and applied to the seeds. Similar plates were prepared for gram seeds (both broken and intact) also. All the treated and untreated plates were incubated at 28 °C for 60 days. The moisture level was maintained by spraying sterilized water at regular interval of ten days. Germination ability of both the treated and untreated seeds was observed after 60 days. Schematic representation of the in-vitro challenge experiment is presented in Fig. 1.

**Figure 14:**
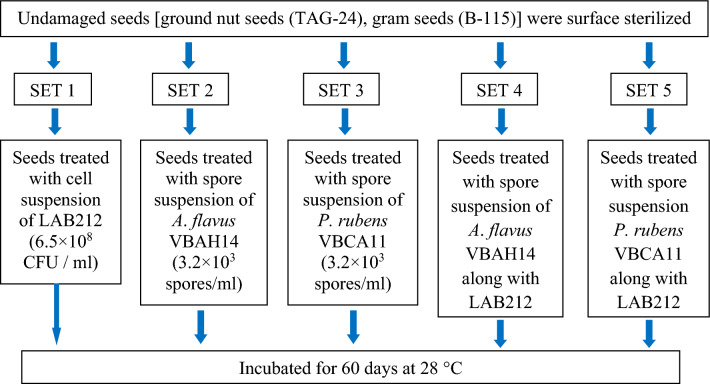
Schematic representation of the in-vitro challenge experiment of undamaged seeds [ground nut seeds (TAG-24), gram seeds (B-115)] using *Limosilactobacillus fermentum* LAB212.

### Evaluation of antioxidant activity by DPPH free radical scavenging assay

Different concentration of the CFS of LAB212 was tested for determining the antioxidant activity using 2, 2-Diphenyl-1-picrylhydrazyl (DPPH) (Sigma-Aldrich) free radical scavenging assay^28^ 100 μl of different concentrations of the methanolic fractions were mixed with 2900 μl DPPH solution and incubated in dark at room temperature for 30 min. In control set instead of using 100 μl methanolic fractions, only 100 μl methanol was used. The absorbance was recorded at 517 nm and the percentage of inhibition (%I) of free radical scavenging activity of methanolic extract was calculated as: %I = [(A_blank_ − A_sample_)/A_blank_] × 100, where, A _blank_: Absorbance of control, A _sample_: Absorbance of methanolic solution and DPPH, IC50 was calculated by plotting straight line equation.

### Formulation of LAB212 for storage

Suitable formulation of LAB212 for storage was standardized by trial and error method. Initially LAB212 was grown in 600 ml MRS broth and harvested by centrifugation at 6000 rpm for 5–6 min at 4 °C. CFS was discarded and cell pellet was re-suspended in 5 mM Na-P buffer. On the other hand *A*. *flavus* VBAH14 and *P*. *rubens* VBCA11 was grown in ME broth and a spore suspension of both the fungal strains were prepared. Carboxymethylcellulose (CMC) and silica were used as sticker molecule and inulin (0.4 g/lit) as prebiotics. Moisture level was maintained spraying sterile water after 20 days interval. MRS and modified MRS (Peptone 5 g/lit, dextrose 10 g/lit, tween80 1 g/lit, ammonium citrate 2 g/lit, sodium acetate 5 g/lit, magnesium sulphate 0.10 g/lit, manganese sulphate 0.05 g/lit, dipotassium hydrogen phosphate 2 g/lit) broth was provided as nutrient supplements. All the experiment sets were observed for 6 months. The whole experiment set was kept in room temperature 26 ± 0.2 °C.

### Data analyses

All the performed experiments were repeated and replicated at least thrice ± SE (standard error) and the collected data were independently observed. The means and standard deviations were calculated using Microsoft Excel 2007 program.

## Data Availability

All the data included in the manuscript are relevant raw data. Supporting reports used during study are given in references. Moreover the study is uploaded in Shodhganga through Visva-Bharati library network (http://hdl.handle.net/10603/400784). The bacterial strain sequence data is deposited at NCBI gene bank with accession number Accession No. MZ359838.

## References

[1] Illikoud N (2019). Genotypic and phenotypic characterization of the food spoilage bacterium *Brochothrix thermosphacta*. Food Microbial..

[2] Footprint, F. W. *Food Wastage Footprint Full-Cost Accounting: Final Report*. (Food Wastage Footprint, 2014).

[3] Tournas VH, Heeres J, Burgess L (2006). Moulds and yeasts in fruit salads and fruit juices. Food Microbial..

[4] Gould GW (1996). Methods for preservation and extension of shelf life. Int. J. Food Microbiol..

[5] Petruzzi, L., Corbo, M. R., Sinigaglia, M. & Bevilacqua, A. Microbial spoilage of foods: Fundamentals. in *The microbiological Quality of Food*, 1–21. (Woodhead Publishing, 2017).

[6] Dalié DKD, Deschamps AM, Richard-Forget F (2010). Lactic acid bacteria: Potential for control of mould growth and mycotoxins: A review. Food Control.

[7] Von Wright, A. & Axelsson, L. Lactic acid bacteria: An introduction. in *Lactic Acid Bacteria*, 1–16. (CRC Press, 2019).

[8] Åvall-Jääskeläinen S, Palva A (2005). *Lactobacillus* surface layers and their applications. FEMS Microbiol. Rev.

[9] Hammes WP (2003). The Prokaryotes: An Evolving Electronic Resource for the Microbiological Community.

[10] Lindgren SE, Dobrogosz WJ (1990). Antagonistic activities of lactic acid bacteria in food and feed fermentations. FEMS Microbiol. Rev..

[11] Lavermicocca P (2000). Purification and characterization of novel antifungal compounds from the sourdough *Lactobacillus plantarum* strain 21B. Appl. Environ. Microb..

[12] Ström K, Sjögren J, Broberg A, Schnürer J (2002). *Lactobacillus plantarum* MiLAB 393 produces the antifungal cyclic dipeptides cyclo (L-Phe-L-Pro) and cyclo (L-Phe-trans-4-OH-L-Pro) and 3-phenyllactic acid. Appl. Environ. Microb..

[13] Magnusson J, Ström K, Roos S, Sjögren J, Schnürer J (2003). Broad and complex antifungal activity among environmental isolates of lactic acid bacteria. FEMS Microbiol. Lett..

[14] Barman S, Ghosh R, Sengupta S, Mandal NC (2017). Longterm storage of post-packaged bread by controlling spoilage pathogens using *Lactobacillus fermentum* C14 isolated from homemade curd. PLoS ONE.

[15] Rouse S, Harnett D, Vaughan A, Sinderen DV (2008). Lactic acid bacteria with potential to eliminate fungal spoilage in foods. J. Appl. Microbiol..

[16] Mandal V, Sen SK, Mandal NC (2007). Detection, isolation and partial characterization of antifungal compound (s) produced by *Pediococcus acidilactici* LAB 5. Nat. Prod. Commun..

[17] De Man JC, Rogosa D, Sharpe ME (1960). A medium for the cultivation of lactobacilli. J. Appl. Microbiol.

[18] Magnusson J, Schnürer J (2001). *Lactobacillus **coryniformis* subsp. *coryniformis* strain Si3 produces a broad-spectrum proteinaceous antifungal compound. Appl. Environ. Microbiol..

[19] Saitou N, Nei M (1987). The neighbor-joining method: A new method for reconstructing phylogenetic trees. Mol. Biol. Evol..

[20] Tamura K (2011). MEGA5: Molecular evolutionary genetics analysis using maximum likelihood, evolutionary distance, and maximum parsimony methods. Mol. Biol. Evol..

[21] Kimura M (1980). A simple method for estimating evolutionary rates of base substitutions through comparative studies of nucleotide sequences. J. Mol. Evol..

[22] Ghosh R, Barman S, Mukherjee R, Mandal NC (2016). Role of phosphate solubilizing *Burkholderia* spp. for successful colonization and growth promotion of *Lycopodium **cernuum* L. (Lycopodiaceae) in lateritic belt of Birbhum district of West Bengal, India. Microbiol. Res..

[23] Suh JW, Lee SH, Chung BC (1997). GC-MS determination of organic acids with solvent extraction after cation-exchange chromatography. Clin. Chem..

[24] Wang YQ, Ye DQ, Zhu BQ, Wu GF, Duan CQ (2014). Rapid HPLC analysis of amino acids and biogenic amines in wines during fermentation and evaluation of matrix effect. Food Chem..

[25] Burton KS (1956). A study of the conditions and mechanism of the diphenylamine reaction for the colorimetric estimation of deoxyribonucleic acid. Biochem. J..

[26] Lowry OH, Rosebrough NJ, Farr AL, Randall RJ (1951). Protein measurement with the Folin phenol reagent. J. Biol. Chem..

[27] Wiseman DW, Marth EH (1981). Growth and aflatoxin production by *Aspergillus parasiticus* when in the presence of *Streptococcus lactis*. Mycopathologia..

[28] Blois MS (1958). Antioxidant determinations by the use of a stable free radical. Nature..

[29] Axel C, Brosnan B, Zannini E, Peyer LC, Furey A, Coffey A, Arendt EK (2016). Antifungal activities of three different *Lactobacillus* species and their production of antifungal carboxylic acids in wheat sourdough. Appl. Microbiol. Biotechnol..

[30] Muhialdin BJ, Hassan Z, Bakar FA, Saari N (2016). Identification of antifungal peptides produced by *Lactobacillus plantarum* IS10 grown in the MRS broth. Food Control..

[31] Reitermayer D, Kafka TA, Lenz CA, Vogel RF (2018). Interrelation between Tween and the membrane properties and high pressure tolerance of *Lactobacillus plantarum*. BMC Microbiol..

[32] Bulgasem BY, Lani MN, Hassan Z, Yusoff WMW, Fnaish SG (2016). Antifungal activity of lactic acid bacteria strains isolated from natural honey against pathogenic *Candida* species. Mycobiology..

[33] Barrios-Roblero C, Rosas-Quijano R, Salvador-Figueroa M, Gálvez-López D, Vázquez-Ovando A (2019). Antifungal lactic acid bacteria isolated from fermented beverages with activity against *Colletotrichum gloeosporioides*. Food Biosci..

[34] Gerez CL, Torres MJ, De Valdez GF, Rollán G (2013). Control of spoilage fungi by lactic acid bacteria. Biol. Control..

[35] Corsetti A, Gobbetti M, Rossi J, Damiani P (1998). Antimould activity of sourdough lactic acid bacteria: Identification of a mixture of organic acids produced by *Lactobacillus sanfrancisco* CB1. Appl. Microbiol. Biotechnol..

[36] Chatterjee S, Ghosh R, Mandal NC (2019). Production of bioactive compounds with bactericidal and antioxidant potential by endophytic fungus *Alternaria alternata* AE1 isolated from *Azadirachta indica* A. Juss. PLoS ONE.

[37] Idziak, E. S., & Coallier-Ascah, J. *Streptococcus lactis* inhibition of aflatoxin production by *Aspergillus flavus*. in *Microbial Associations and Interactions in Food: Proceedings of the 12th International IUMS-ICFMH Symposium* 1983 (ed by. Kiss, I., Deak, T., Incze, K. & Reidel, D.) (1984).

[38] Peltonen K, El-Nezami H, Haskard C, Ahokas J, Salminen S (2001). Aflatoxin B1 binding by dairy strains of lactic acid bacteria and bifidobacteria. J. Dairy Sci..

[39] Achal V, Savant VV, Reddy MS (2007). Phosphate solubilization by a wild type strain and UV-induced mutants of *Aspergillus tubingensis*. Soil Biol. Biochem..

[40] Kumar A, Mathimaran A, Shrikanta AH, Govindaswamy V (2018). Role of partially saturated canthaxanthin and ergosterol in the survival of *Aspergillus carbonarius* mutant at extreme acidic condition. Microbiology.

[41] Kumpulainen JT, Salonen JT (1999). Natural Antioxidants and Anticarcinogens in Nutrition, Health and Disease (No. 240).

[42] Halliwell B (1994). Free radicals, antioxidants, and human disease: Curiosity, cause, or consequence?. Lancet.

